# The C‐Reactive Protein–Triglyceride–Glucose Index and Functional Outcomes in Acute Ischemic Stroke Patients Following Endovascular Thrombectomy

**DOI:** 10.1002/brb3.71770

**Published:** 2026-09-29

**Authors:** Jing Bian, Junfeng Xu, Xiuyun Li, Xianhui Ding, Yapeng Guo, Ke Yang, Xianjun Huang, Shoucai Zhao, Zhiming Zhou, Zibao Li

**Affiliations:** ^1^ Department of Neurology Yijishan Hospital of Wannan Medical University Wuhu Anhui China

**Keywords:** 90‐day functional outcomes, acute ischemic stroke, C‐reactive protein‐triglyceride glucose index, endovascular thrombectomy

## Abstract

**Background and Purpose:**

The C‐reactive protein–triglyceride–glucose index (CTI), capturing insulin resistance (IR) and systemic inflammation, is related to stroke prognosis in general, but has not yet been studied with respect to functional outcomes after endovascular thrombectomy (EVT).

**Methods:**

A retrospective analysis was carried out on individuals with acute ischemic stroke (AIS) experiencing EVT (September 2018–December 2024). CTI is measured as: 0.412 × Ln (C‐reactive protein) (mg/L) + Ln [fasting triglyceride (mg/dL) × fasting glucose (mg/dL)]/2. The endpoint was unfavorable functional outcomes, characterized by a modified Rankin Scale (mRS) score of 3–6 at 90‐day follow‐up. Restricted cubic splines (RCS) and logistic regression (LR) analysis were utilized to estimate the link between the CTI and clinical outcomes.

**Results:**

Among 813 patients, a high CTI level was significantly correlated with unfavorable functional outcome (*p* < 0.05). Compared with patients in the lowest quartile (Q1) of CTI, those in the highest CTI quartile (Q4) demonstrated an increased likelihood of unfavorable outcome (adjusted odds ratio [aOR] of 2.04, 95% confidence interval (95% CI) of 1.17–3.54). RCS modeling, after adjustment for significant variables identified in the univariate analysis, confirmed that there was no nonlinear connection between CTI levels and the probability of unfavorable functional recovery. Stratified analysis indicated that these associations were more significant among elderly and non‐smoking patients.

**Conclusion:**

The CTI is an independent indicator of functional outcomes in individuals with AIS undergoing EVT, underlining its potential clinical value as a readily accessible marker for risk stratification and individualized prognostic assessment.

## Graphical abstract

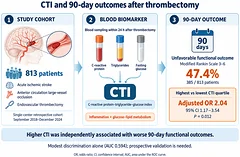

## 1 Introduction

Acute ischemic stroke (AIS) derived from large vessel occlusion (LVO) is distinguished by rapid onset and severe condition, with increased rates of disability and mortality, posing a heavy burden on society and patients (Tu et al. 2021). Endovascular thrombectomy (EVT), as a successful treatment for LVO‐AIS, can rapidly restore perfusion to ischemic brain tissue and significantly improve patient outcomes (Jadhav et al. 2021). Nevertheless, approximately 50% of individuals with effective recanalization after EVT still had persistent functional dependency during follow‐up (X. Huang, Ding, et al. 2025). Hence, performing an in‐depth exploration of the factors influencing functional recovery has become a crucial focus of current research.

Insulin resistance (IR) is a pathophysiological condition distinguished by decreased insulin sensitivity and elevated circulating insulin levels (Lu et al. 2023; Yang et al. 2023). The method that integrates glucose and triglyceride (TG) metabolism is widely recognized as an economical, efficient, straightforward, and reliable alternative biomarker for IR (Huo et al. 2025). Investigations have indicated that the triglyceride‐glucose index (TyG) level is linked to the outcome of individuals undergoing EVT (Y. Huang, Nie, et al. 2025; Sun et al. 2024; Wang et al. 2025), while a small subset of studies has detected that the TyG levels and the outcome of the same therapy did not significantly correlate (Lee et al. 2021; Li et al. 2025). Furthermore, previous investigations have illustrated that IR correlates positively with inflammatory markers and influences patient clinical outcomes through inflammation (Y. Huang, Nie, et al. 2025). This indicates that relying solely on a single biomarker may result in a true risk underestimation. In clinical settings, C‐reactive protein (CRP), as an inflammatory marker, is also frequently employed to evaluate stroke severity and forecast patient outcomes (Ji et al. 2022); these markers are similarly relevant for individuals with AIS who are undergoing EVT (Yeh et al. 2023). Therefore, integrating comprehensive assessments of IR and inflammatory status may provide prognostic information for the prediction of outcomes in individuals with ischemic stroke underwent EVT.

The C‐reactive protein–triglyceride–glucose index (CTI) was first suggested by Ruan et al. (2022), holding significant value in predicting the occurrence, progression, and outcome of stroke in ordinary individuals in earlier investigations (Huo et al. 2025). Nonetheless, the connection between the CTI and clinical outcomes in individuals with AIS experiencing EVT is still unclarified.

## 2 Patients and Methods

### 2.1 Patients Selection

This investigation constitutes a retrospective cohort analysis. Consecutive enrollment included individuals treated with EVT for AIS secondary to anterior circulation LVO at the Neurology Department, The First Affiliated Hospital of Wannan Medical University (September 2018–December 2024). The selection criteria were as follows: (1) aged 18 years or more; (2) an onset‐to‐puncture time (OTP) not exceeding 24 h; (3) a pre‐stroke modified Rankin Scale (mRS) score below 2; and (4) confirmed occlusion in either the internal carotid artery (ICA) or the M1 segment of the middle cerebral artery (MCA). Exclusion criteria comprised (1) cases with multiple vessel occlusions (MVO), M2 segment MCA or anterior cerebral artery (ACA) occlusions; (2) absence of blood test data for CRP, TG, or fasting blood glucose (FBG); and (3) missing 90‐day follow‐up data.

The Ethical Review Board of Yijishan Hospital (20190039) gave its approval to this investigation, and informed consent was acquired from each participant or his or her legally authorized representative.

### 2.2 Clinical Data Collection

The collection of demographic and clinical data (e.g., age, gender, medical history, occlusion site, stroke subtypes, radiological findings, procedural details, and laboratory measurements) was conducted retrospectively from prospectively registered electronic medical records. Stroke severity was estimated via the National Institutes of Health Stroke Scale (NIHSS) score (Brott et al. 1989), and we classified stroke subtypes per the Trial of Org 10172 in Acute Stroke Treatment (TOAST) (Adams et al. 1993). Effective recanalization was considered as a Thrombolysis in Cerebral Infarction (mTICI) score of 2b or 3 (Yoo et al. 2013). Before emergency EVT, we utilized digital subtraction angiography (DSA) to perform retrograde angiography of the occluded vessel. This procedure evaluates the status of collateral circulation. The collateral circulation grading system is as follows: Grade 0 indicates minimal or absent collateral blood flow in the affected area or occurs when the occluded vessel's territory exceeds two‐thirds of the collateral compensation capacity. Grade 1 indicates collateral perfusion covering between one‐third and two‐thirds of the occluded vessel's territory. Grade 2 indicates collateral perfusion covering more than two‐thirds of the territory or direct communication between collateral vessels and the proximal segment of the primary vessel (Ji et al. 2022).

### 2.3 Blood Sample Measurements

We collected fasting blood samples within 24 h post‐EVT and examined them using standard laboratory procedures. CRP was assayed by immunoturbidimetry, TG by the glycerol phosphate oxidase‐peroxidase (GPO‐PAP) technique, and FBG by the glucose oxidase method. All measurements were performed on the Beckman Coulter AU 5800 automatic biochemical analyzer. The TyG was measured as Ln [TG (mg/dL) × FBG (mg/dL)] divided by 2 (Y. Huang, Nie, et al. 2025; Ruan et al. 2022). The CTI was measured as CTI = 0.412 × Ln (CRP (mg/L)) + TyG (Huo et al. 2025; Ruan et al. 2022; Yang and Liu 2025).

### 2.4 Outcomes

Functional outcomes were evaluated by another stroke neurologist through scheduled in‐person consultations or telephonic follow‐ups. The clinical outcomes are estimated using the mRS 90 days following stroke onset, with scores of 0–2 suggesting favorable functional outcomes, whereas scores of 3 or higher indicate unfavorable outcomes and scores of 6 denote death.

### 2.5 Statistical Analysis

To minimize analytical bias stemming from missing data, we excluded all participants with incomplete variable records from the final study cohort.

Continuous variables were represented as median [interquartile range (IQR)] or mean ± standard deviation (SD). Categorical variables were reported as count (%). Comparisons between continuous variables were conducted using *t*‐tests for data with normal distribution and Mann–Whitney *U* tests (2 groups) or Kruskal–Wallis *H* tests (several groups) for data with abnormal distribution, while we utilized chi‐square or Fisher's exact tests for comparisons between categorical variables. We conducted univariate analysis to identify significant factors related to the outcomes. These variables were subsequently incorporated into binary multivariate logistic regression (LR) analysis to estimate the impact of the CTI on the outcomes, and adjusted odds ratios (aOR) and matched 95% confidence intervals (CIs) were documented.

Nonlinear links between CTI and clinical outcomes were estimated via restricted cubic splines (RCS), and the significance of the non‐linear model compared to the linear model was detected via likelihood ratio tests. Additionally, receiver operating characteristic (ROC) curves were created to estimate the CTI's predictive value for poor outcomes. Furthermore, stratified analyses were conducted to examine potential modifying influences of demographic and past medical history on the correlation between clinical outcomes and CTI. To validate the strength of the final predictors, sensitivity analyses were performed by repeating multivariate models with a stricter significance threshold (*p* < 0.01) in the univariate screening stage.

IBM SPSS Statistics (v26.0), R (v4.3.1), and MedCalc (v20.1.0) were employed for statistical analyses, with significance set at *p*<0.05.

## 3 Results

### 3.1 Baseline Features

Following strict exclusion criteria application, we included 813 eligible individuals in the final analysis, while excluded 349 individuals (Figure 1). We compared demographic features and baseline NIHSS between included and excluded individuals. No significant variations were detected (*p* > 0.05) (Table S1). Table 1 depicts the comprehensive baseline demographic and clinical parameters.

**FIGURE 1:**
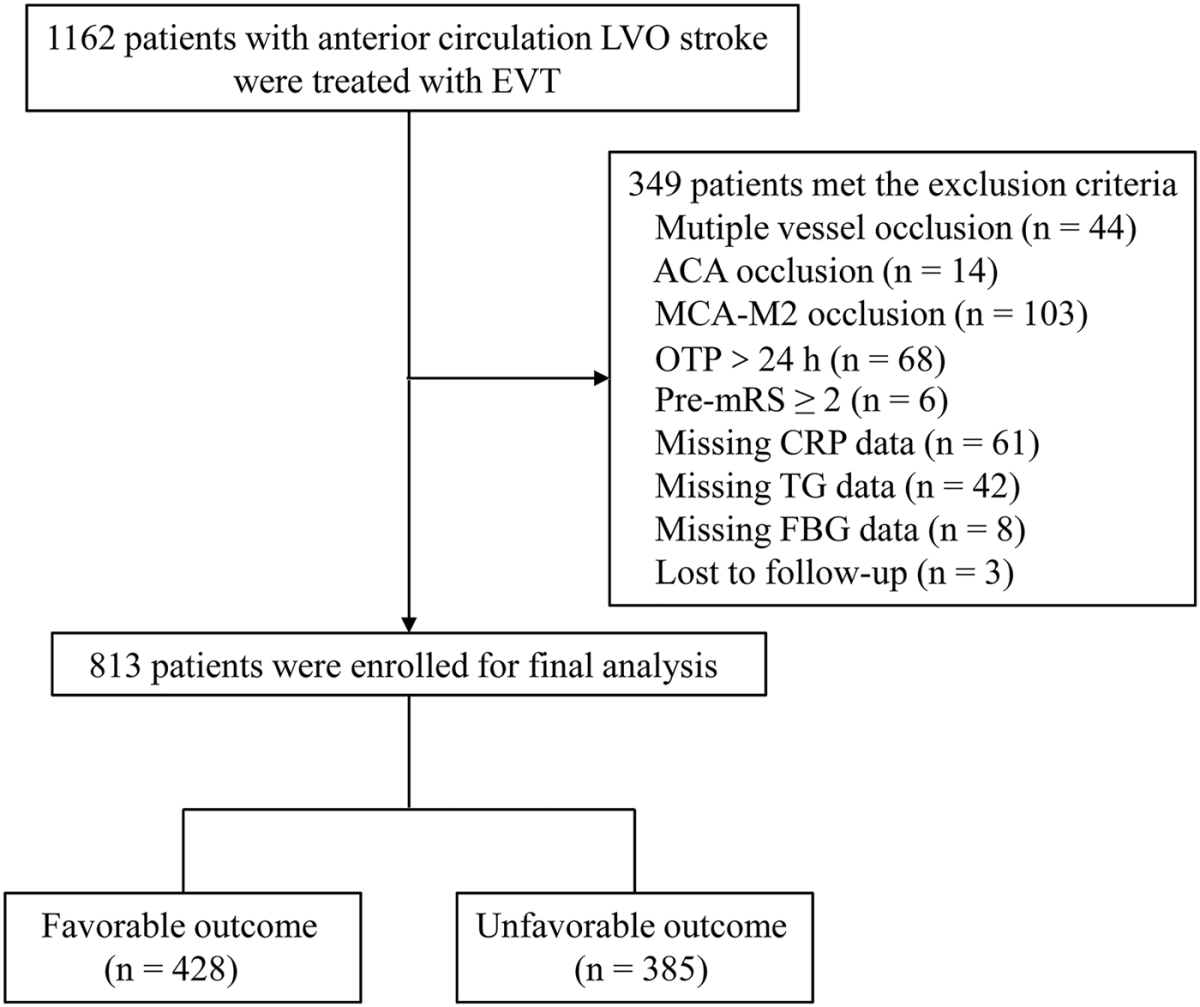
Flowchart of the study population. ACA, anterior cerebral artery; CRP, C‐reactive protein; EVT, endovascular thrombectomy; FBG, fasting blood glucose; LVO, large vessel occlusion; MCA, middle cerebral artery; mRS, modified Rankin Scale; OTP, onset‐to‐puncture time; TG, triglyceride.

**TABLE 1:** Baseline features among groups classified by the outcome.

Characters	Favorable outcome (*n* = 428)	Unfavorable outcome (*n* = 385)	*p* value
Age, median (IQR), years	68 (58,75)	73 (65, 78.50)	<0.001
Male, *n* (%)	286 (66.82)	210 (54.55)	< 0.001
Medical history, *n* (%)			
History of smoking	170 (39.72)	98 (25.45)	< 0.001
History of drinking	129 (30.14)	92 (23.90)	0.046
Hypertension	254 (59.35)	264 (68.57)	0.006
Diabetes mellitus	64 (14.95)	70 (18.18)	0.215
Atrial fibrillation	173 (40.42)	232 (60.26)	< 0.001
Clinical data			
Baseline systolic blood pressure, mean ± SD, mmHg	158 (137.25, 168)	155 (140, 172.50)	0.003
Baseline diastolic blood pressure, mean ± SD, mmHg	85 (77, 94)	86 (77,95)	0.700
Baseline NIHSS, median (IQR)	12 (10,15)	15 (12,18)	< 0.001
Intravenous thrombolysis, *n* (%)	55 (12.85)	36 (9.35)	0.114
Occlusion site, *n* (%)			0.001
Internal carotid artery	163 (38.08)	191 (49.61)	
Middle cerebral artery‐M1	265 (61.92)	194 (50.39)	
TOAST type, *n* (%)			< 0.001
Large artery atherosclerosis	167 (39.02)	91 (23.64)	
Cardioembolism	205 (47.90)	251 (65.19)	
Others or undetermined	56 (13.08)	43 (11.17)	
Radiological findings and procedure process			
Baseline Alberta Stroke Program Early CT, median (IQR)	9 (8, 10)	8 (5,9)	< 0.001
Onset‐to‐puncture time, median (IQR), min	317 (240, 450)	310 (240, 455)	0.624
Onset‐to‐reperfusion time, median (IQR), min	376 (285, 500.50)	380 (301, 537)	0.103
Collateral, *n* (%)			< 0.001
Grade 0	19 (4.44)	93 (24.16)	
Grade 1	92 (21.50)	121 (31.43)	
Grade 2	317 (74.07)	171 (44.42)	
mTICI (2b/3), *n* (%)	419 (97.90)	345 (89.61)	< 0.001
Laboratory measurements			
Triglyceride‐glucose index, median (IQR)	4.55 (4.36, 4.77)	4.63 (4.43, 4.85)	0.001
CTI, median (IQR)	5.42 (5.01, 5.79)	5.64 (5.14, 6.07)	<0.001

*Note*: Continuous data are summarized using either mean ± SD or median (IQR), while categorical data are represented as *n* (%). For comparing continuous variables, an independent‐samples *t*‐test or a Mann–Whitney *U* test was conducted, while the chi‐square test was utilized for categorical variables.

Abbreviations: CTI, C‐reactive protein‐triglyceride‐glucose index; IQR, interquartile range; NIHSS, National Institutes of Health Stroke Scale; mTICI, modified thrombolysis in cerebral infarction; TOAST, Trial of Org 10172 in Acute Stroke Treatment.

The individuals were classified into two distinct groups depending on their functional outcomes: 428 individuals (52.6%) demonstrated favorable clinical recovery, whereas 385 patients (47.4%) exhibited poor outcomes, among whom 136 patients died. In univariate analysis of patient data, the CTI differed significantly between groups (median 5.64 vs. 5.42; *p* < 0.001). Other relevant variables comprised older age, male sex, history of hypertension, baseline systolic blood pressure (SBP), drinking, baseline NIHSS score, smoking history, occlusion site, atrial fibrillation, TOAST classification, baseline ASPECTS, collateral, mTICI (2b/3), and the TyG (Table 1).

Multivariable LR analysis considered CTI as an independent risk factor for unfavorable outcomes, demonstrating an aOR of 1.45 (95% CI: 1.06–2.00). Other risk factors include age, smoking history, NIHSS score, ASPECT score, collateral, and mTICI (2b/3) (Table S2). Furthermore, multivariable regression analysis demonstrated that CTI also acted as an independent indicator of all‐cause mortality at 90 days (aOR = 2.18, *p* < 0.001) (Table S3).

RCS analysis established no nonlinear link between CTI levels and the probability of poor functional recovery following adjustment for covariates with statistical significance in univariate analysis (*p* for nonlinearity = 0.385) (Figure 2).

**FIGURE 2:**
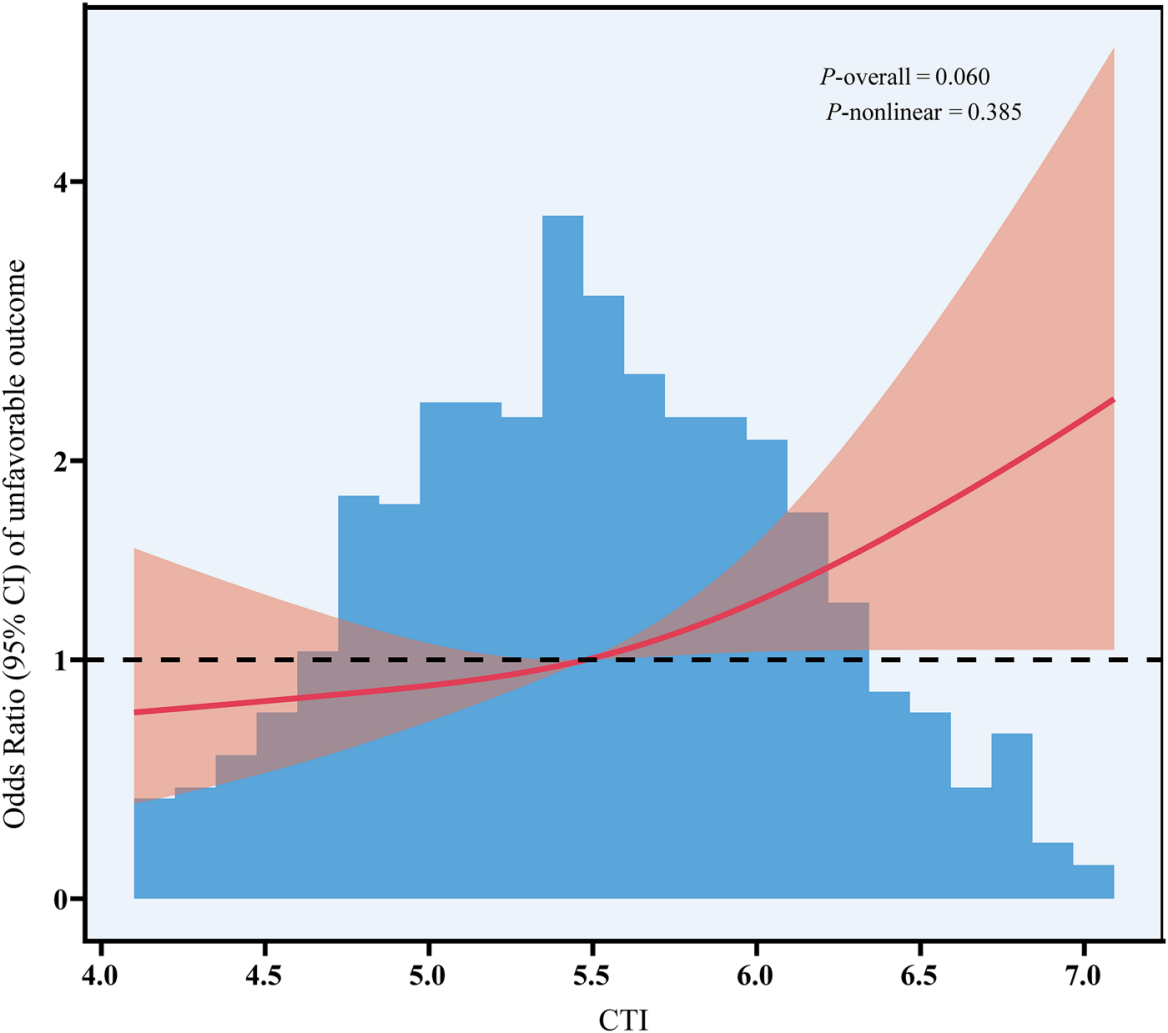
Association between CTI and 90‐day unfavorable outcome with the restricted cubic spline function. Adjusted for age, male sex, smoking history, history of drinking, hypertension, atrial fibrillation, baseline systolic blood pressure (SBP), baseline NIHSS score, occlusion site, TOAST classification, baseline ASPECTS, collateral, mTICI (2b/3), and the TyG. CTI, C‐reactive protein‐triglyceride‐glucose index.

### 3.2 ROC Curve Analysis

The ROC curve was utilized to determine CTI's predictive ability for unfavorable outcomes and its predictive value. The ROC analysis illustrated an AUC of 0.594 (95% CI 0.554–0.663, *p* < 0.001), and the maximum predictive efficiency was achieved when the cutoff value of CTI was 5.60 (Youden's index 0.179). This threshold provided 55.2% sensitivity and 65.7% specificity for predicting unfavorable outcome (Figure S1). Furthermore, to evaluate whether the TyG affects the predictive capability of the CTI‐established model for adverse outcomes, we compared two predictive models: one including the TyG index and the other excluding it. The comparison of ROC curve analysis illustrated that the AUC of the model with the TyG did not significantly change the predictive value for adverse outcomes compared with that of the model without TyG (*p* > 0.05) (Figure S2).

### 3.3 CTI in Quartiles

All individuals were categorized into four groups depending on the CTI quartiles (Q1: CTI ≤ 5.07; Q2: 5.07< CTI ≤ 5.48; Q3: 5.48< CTI ≤ 5.94; Q4: CTI > 5.94). Individuals in higher CTI quartiles had different demographic and clinical parameters, such as increased prevalence of hypertension, diabetes mellitus, decreased prevalence of atrial fibrillation, more representation of large artery atherosclerosis etiology, elevated levels of admission SBP and NIHSS, and an elevated proportion of unfavorable outcomes (Table S4). Figure 3 depicts the mRS scores’ distribution at three months, ranked by CTI quartiles. We detected a significant shift toward unfavorable functional outcomes among individuals in higher CTI quartiles (Figure 3).

**FIGURE 3:**
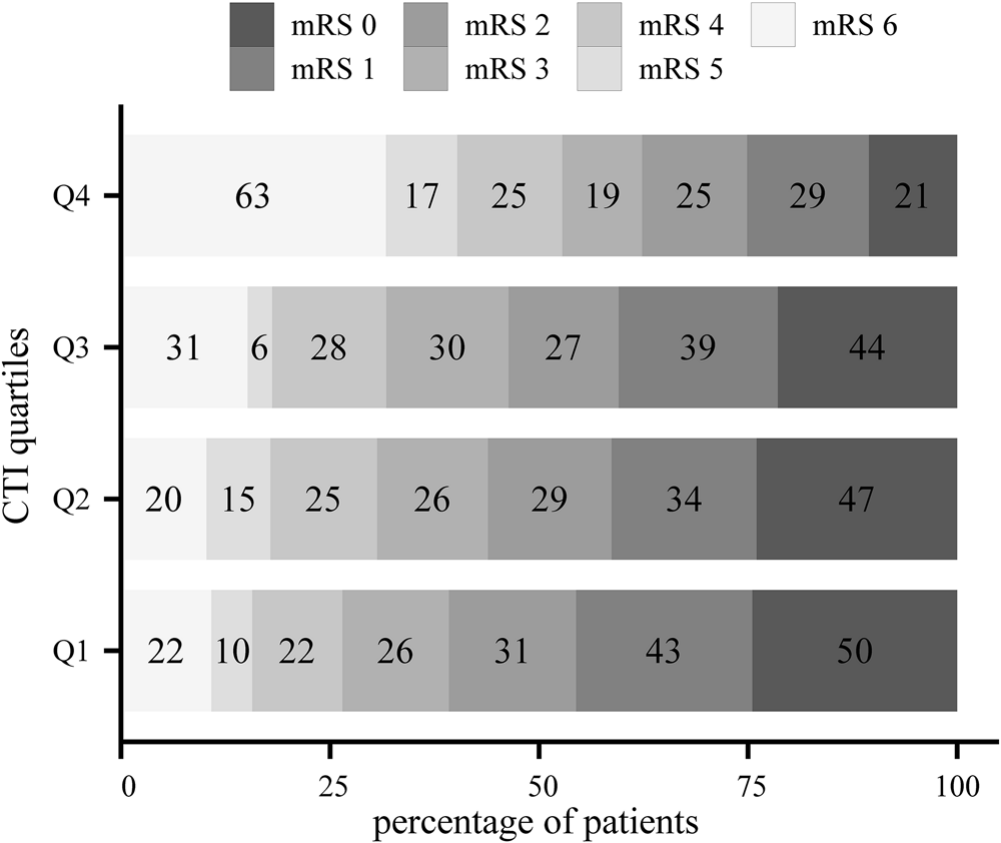
Distribution of modified Rankin Scale scores at 90 days classified by quartiles of CTI. CTI, C‐reactive protein‐triglyceride‐glucose index; mRS, modified Rankin Scale.

### 3.4 LR Analyses (Univariate and Multivariate)

To further examine the link between CTI and poor clinical outcomes, univariate LR analysis was conducted. The outcomes indicated that individuals in the Q4 of CTI showed a significantly elevated risk of unfavorable functional outcomes comparative to the Q1, with OR of 2.56 (95% CI: 1.72–3.83, *p* < 0.001). Post‐adjustment for potential confounders (e.g., gender, age, smoking history, alcohol intake, hypertension, atrial fibrillation, admission SBP, baseline NIHSS, occlusion site, TOAST classification, ASPECTS, collateral circulation status, mTICI (2b/3), and TyG), the association remained significant. Specifically, the aOR for unfavorable outcomes in the Q4 of CTI was 2.04 (95% CI: 1.17–3.54, *p* = 0.012) (Table 2).

**TABLE 2:** Univariate and multivariate LR analyses for CTI subgroup levels and unfavorable outcome.

CTI	Unadjusted OR (95% CI)	*p* value	Adjusted OR (95% CI)	*p* value
Quartile group				
Q1	Reference	Reference	Reference	Reference
Q2	1.12 (0.78–1.66)	0.573	1.31 (0.81–2.10)	0.268
Q3	1.34 (0.90–1.98)	0.146	1.40 (0.85–2.30)	0.181
Q4	2.56 (1.72–3.83)	< 0.001	2.04 (1.17–3.54)	0.012
By optimal cut‐off derived from ROC curve				
CTI < 5.60	Reference	Reference	Reference	Reference
CTI ≥ 5.60	2.00 (1.51‐2.66)	< 0.001	1.76 (1.21–2.56)	0.003

*Note*: Adjusted for age, male sex, smoking history, history of drinking, hypertension, atrial fibrillation, baseline SBP, baseline NIHSS score, occlusion site, TOAST classification, baseline ASPECTS, collateral, mTICI (2b/3), and the TyG.

Abbreviation: CTI, C‐reactive protein‐triglyceride‐glucose index.

Furthermore, individuals were grouped into two groups as per the optimal cutoff value of CTI (5.60) determined by ROC curve analysis. Individuals with CTI ≥ 5.60 had a significantly elevated risk of unfavorable outcomes comparative to those with CTI < 5.60, yielding an unadjusted OR of 2.00 (95% CI: 1.51–2.66, *p* < 0.001). Following further adjusting for the aforementioned covariates, the risk remained elevated in the CTI ≥ 5.60 group, with an aOR of 1.76 (95% CI: 1.21–2.56, *p* = 0.003) (Table 2).

### 3.5 Subgroup Analyses

We conducted subgroup analyses to estimate potential interactions between various demographic and clinical factors and the connection between the CTI and outcome. Figure 4 illustrates the subgroup analysis results pertaining to the endpoint. Within the subgroup aged ≥ 60 years and without history of smoking, elevated CTI levels were significantly correlated with functional outcomes (Figure 4).

**FIGURE 4:**
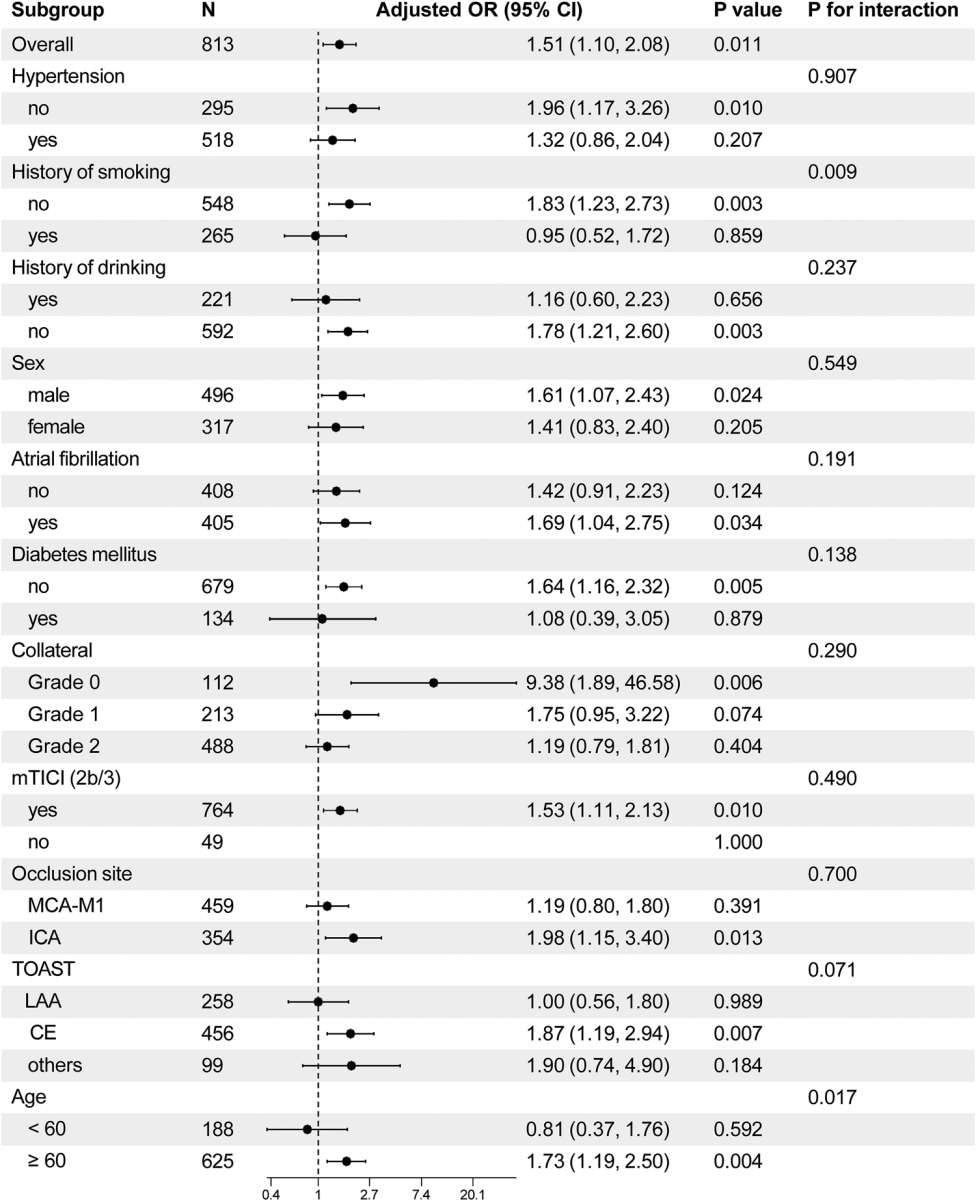
Subgroup and interaction analysis of the link between CTI and clinical outcomes. Adjusted for age, sex, smoking history, history of drinking, hypertension, atrial fibrillation, baseline systolic blood pressure (SBP), baseline NIHSS score, occlusion site, TOAST classification, baseline ASPECTS, collateral, mTICI (2b/3), and the TyG. CE, cardioembolism; CTI, C‐reactive protein‐triglyceride‐glucose index; ICA, internal carotid artery; LAA, large artery atherosclerosis; MCA, middle cerebral Artery; mTICI, modified thrombolysis in cerebral infarction; TOAST, the Trial of Org 10172 in Acute Stroke Treatment.

### 3.6 Sensitivity Analyses

Sensitivity analysis was conducted by incorporating variables with *p* < 0.01 from the univariate into the multivariate LR model. The outcomes aligned with the main analysis and confirmed that CTI was independently associated with unfavorable outcomes (Table S5).

## 4 Discussion

Our findings suggest that a higher CTI is independently linked to poorer functional outcomes at 90 days in individuals with AIS receiving EVT treatment. Higher CTI levels show no nonlinear relationship with worse functional outcomes. Notably, this correlation is stronger in elderly and non‐smoking patients.

Chen et al. (2026) screened 873 candidates receiving EVT and included 493 eligible patients. Their research confirmed that CTI independently predicted 90‐day poor functional outcomes (aOR = 1.47) (Chen et al. 2026). In a single‐center retrospective analysis of 366 individuals with EVT published by Zhou et al. (2026), stratification based on the optimal CTI threshold revealed a significant connection between increased CTI values and higher risks of adverse clinical outcomes. Consistent with established literature, our investigation enrolled an enlarged cohort comprising 813 individuals. Following adjustment for confounding variables, continuous CTI demonstrated an independent link to adverse outcomes at 90 days (aOR = 1.45) (Chen et al. 2026; Zhou et al. 2026). Furthermore, categorizing CTI based on the optimal cutoff point and quartile divisions also revealed significant associations with unfavorable outcomes.

Multiple investigations have demonstrated that the TyG, serving as an alternative marker for IR, exhibits a significant correlation with adverse functional outcomes in individuals with AIS following EVT (Y. Huang, Nie, et al. 2025; Sun et al. 2024; Wang et al. 2025). However, a few investigations have illustrated that the TyG is not significantly related to the outcome of individuals experiencing EVT (Lee et al. 2021; Li et al. 2025). This investigation did not detect a link between the TyG levels and the clinical outcomes of individuals undergoing EVT. This may be linked to the investigation's population and limitations of a single indicator. Additionally, studies have shown that TyG alone does not significantly correlate with stroke outcome. However, when TyG is combined with other indicators (e.g., inflammatory markers and metabolic markers) the correlation becomes significant (Y. Huang et al. 2024; Li et al. 2025). Remarkably, inflammation may have a mediating function in the link between IR and stroke prognosis. Huang et al. found that among 1305 AIS patients who underwent EVT, those in the highest TyG quartile exhibited a significantly elevated functional dependency risk (aOR 1.79). Furthermore, inflammation partially explained this association (Y. Huang, Nie, et al. 2025). This aligns with our research hypothesis that the CTI integrates two key pathophysiological processes, inflammation and metabolic disorder, and may offer greater predictive value than the TyG index alone.

Despite the relatively low AUC value, it is crucial to recognize that the CTI forms part of a broader predictive model, and when integrated with other clinical and biomarker data, the model's performance may improve (Yang and Liu 2025). Sun et al. found that among 424 individuals with LVO‐AIS who experienced EVT, an increased TyG was linearly correlated with poorer 90‐day functional outcomes (mRS > 2) (Sun et al. 2024). This discovery reinforces our research findings. Furthermore, no nonlinear link was found between CTI levels and the outcomes of patients undergoing EVT after multivariate adjustments, suggesting that as the CTI value increases, the risk of a poor outcome rises.

Regarding the outcomes of subgroup analysis, our investigation demonstrated that the connection between CTI and adverse outcome was more noticeable in elderly individuals (≥ 60 years) than in younger individuals (< 60 years). This finding may be attributed to the prevalence of chronic low‐grade inflammatory states and metabolic disorders among elderly patients (L.‐Y. Huang, Liu, et al. 2023). The immune function in elderly patients declines, and they often have multiple comorbidities (Finger et al. 2022). These factors may act synergistically to exacerbate the negative impact of inflammation and IR on stroke outcome (Wang et al. 2025). The correlation between CTI levels and poor prognosis in EVT was more pronounced in non‐smoking patients. This may be attached to smoking itself, as a potent factor of inflammation and oxidative stress (Fenercioglu et al. 2025), which obscures the prognostic predictive value of traditional metabolic and inflammatory markers. Conversely, the prognostic disparities in non‐smoking patients are more reliant on baseline levels of underlying inflammation and glucose‐lipid metabolism.

CTI represents a convergence of IR and inflammatory processes, serving as a dual indicator of metabolic dysregulation in glucose/lipid metabolism and systemic inflammation—two core pathological drivers of cerebrovascular events (Zhou et al. 2025). IR fosters a prothrombotic environment by amplifying platelet activation and compromising endothelial integrity (Saltiel and Olefsky 2017), perpetuating vascular inflammation and endothelial dysfunction (Lteif et al. 2005). In ischemia‐reperfusion injury, experimental models of IR demonstrate that disrupted glucose metabolism and upregulated inflammatory mediators play mechanistic roles (Apaijai et al. 2014). As an acute‐phase protein, CRP elevation signals active inflammatory states (Yeh et al. 2023). Stroke patients receiving EVT typically exhibit LVO with significant perfusion deficits, where ischemic tissue triggers robust systemic inflammation through cytokine release. These inflammatory agents intensify neuroinflammation, accelerating infarct expansion (Jayaraj et al. 2019). A reciprocal relationship exists between metabolic dysfunction and inflammation in stroke pathogenesis. Chronic metabolic disturbances establish a proinflammatory background that amplifies ischemic inflammation, while acute‐phase responses to cerebral ischemia aggravate preexisting metabolic disorders (Saltiel and Olefsky 2017). This complex interaction renders CTI a clinically meaningful composite biomarker that integrates metabolic‐inflammatory parameters while mutually correcting measurement biases and confounding factors of individual indicators, effectively predicting poor prognosis in stroke patients receiving endovascular therapy.

### 4.1 Limitations

This investigation faces notable limitations. First, the retrospective analysis carries inherent risks of selection bias, which may limit how well the studied patient cohort reflects the broader AIS population undergoing EVT treatment. As an observational investigation, it cannot definitively verify causality between CTI and functional outcomes in EVT‐treated AIS. This single‐center study limits the generalizability of our outcomes, and data‐driven covariate selection may increase the risk of false‐positive outcomes. Second, stress‐induced hyperglycemia could also influence our research results. Although we attempted to measure FBG levels shortly after patient admission, we could not guarantee a true fasting state. Furthermore, hyperglycemic reactions are related to larger areas of stroke, which may be a consequence of the stroke rather than a causative factor. Third, based on the TOAST etiological classification, most patients in this study were classified as cardiogenic embolism. However, large artery atherosclerotic infarction is closely related to metabolic comorbidities (e.g., obesity, hypertension, dyslipidemia, diabetes, and IR). Future research involving a larger sample size is necessary. In summary, verifying the correlation between CTI and 90‐day functional outcomes in individuals post‐EVT treatment necessitates conducting a comprehensive multicenter investigation with a larger sample size and a prospective design.

## 5 Conclusion

Our study indicates a correlation between IR and inflammation, assessed by CTI, and 90‐day mRS in individuals with AIS undergoing EVT for LVO. CTI is an easily accessible biomarker in clinical practice that can improve the accuracy of clinical decision‐making by assisting prediction and risk stratification. Upcoming investigations should aim to prospectively validate the correlation between CTI and outcomes following EVT.

## Author Contributions

Conceptualization: **Xiuyun Li** and **Yapeng Guo**. Data curation: Yapeng Guo and **Ke Yang**. Formal analysis: **Zibao Li** and **Shoucai Zhao**. Investigation: **Junfeng Xu** and **Jing Bian**. Software: Junfeng Xu and **Xianhui Ding**. Supervision: **Xianjun Huang** and Shoucai Zhao. Project administration: Xianjun Huang and Zibao Li. Validation: Ke Yang and **Zhiming Zhou**. Visualization: Xiuyun Li and Ke Yang. Writing – original draft: Jing Bian. Writing – review and editing: Zhiming Zhou and Zibao Li.

## Funding

This investigation received funds from the Natural Science Research Project of Universities of Anhui Province in China (2022AH051244), Key Research Project of Wannan Medical College (WK2023ZZD21), Young and middle‐aged faculty members at Wannan Medical University (WK2023ZQNZ39), and Wuhu Science and Technology Project (2023JC28).

## Ethics Statement

The Ethics Committee of Yijishan Hospital, Wannan Medical University reviewed and gave its approval to the protocol. Before study participation, we acquired informed consent from each participant or their legal representative when participants lacked the capacity to provide consent. All research procedures were conducted as per internationally recognized ethical guidelines, including the ethical principles set forth in the Helsinki Declaration.

## Consent

The authors have nothing to report.

## Conflicts of Interest

The authors declare no conflicts of interest.

## Supporting information




**Supplementary Material**: brb371770‐sup‐0001‐SuppMat.docx

## Data Availability

The data that support the findings of this study are available upon reasonable request.

## References

[brb371770-bib-0001] Adams, H. P., Jr. , B. H. Bendixen , L. J. Kappelle , et al. 1993. “Classification of Subtype of Acute Ischemic Stroke. Definitions for Use in a Multicenter Clinical Trial. TOAST. Trial of Org 10172 in Acute Stroke Treatment.” Stroke 24: 35–41. 10.1161/01.str.24.1.35 7678184

[brb371770-bib-0002] Apaijai, N. , S. C. Chattipakorn , and N. Chattipakorn . 2014. “Roles of Obese‐Insulin Resistance and Anti‐Diabetic Drugs on the Heart With Ischemia‐Reperfusion Injury.” Cardiovascular Drugs and Therapy 28: 549–562. 10.1007/s10557-014-6553-6 25283086

[brb371770-bib-0003] Brott, T. , H. P. Adams Jr., C. P. Olinger , et al. 1989. “Measurements of Acute Cerebral Infarction: A Clinical Examination Scale.” Stroke 20: 864–870. 10.1161/01.str.20.7.864 2749846

[brb371770-bib-0004] Chen, W. , X. Bai , T. Wang , L. Jiao , L. Zhang , and H. Li . 2026. “Association of the C‐Reactive Protein–Triglyceride–Glucose Index With Stroke–Heart Syndrome and Clinical Prognosis in Patients Undergoing Endovascular Treatment.” Journal of Cardiovascular Development and Disease 13, no. 5: 179. 10.3390/jcdd13050179 PMC13207347 42188065

[brb371770-bib-0005] Fenercioglu, A. K. , H. Uzun , and D. O. Unal . 2025. “The Convergent Immunopathogenesis of Cigarette Smoke Exposure: From Oxidative Stress to Epigenetic Reprogramming in Chronic Disease.” International Journal of Molecular Sciences 27, no. 1: 187. 10.3390/ijms27010187 PMC12785566 41516068

[brb371770-bib-0006] Finger, C. E. , I. Moreno‐Gonzalez , A. Gutierrez , J. F. Moruno‐Manchon , and L. D. McCullough . 2022. “Age‐Related Immune Alterations and Cerebrovascular Inflammation.” Molecular Psychiatry 27: 803–818. 10.1038/s41380-021-01361-1 PMC9046462 34711943

[brb371770-bib-0007] Huang, L.‐Y. , C.‐H Liu , F.‐Y Chen , C. H Kuo , P Pitrone , and J.‐S. Liu . 2023. “Aging Affects Insulin Resistance, Insulin Secretion, and Glucose Effectiveness in Subjects With Normal Blood Glucose and Body Weight.” Diagnostics 13, no. 13: 2158. 10.3390/diagnostics13132158 PMC10341091 37443552

[brb371770-bib-0008] Huang, X. , X. Ding , H. Wang , et al. 2025. “Magnitude of Systolic Blood Pressure Reduction Following Endovascular Treatment and Clinical Outcomes in Acute Large Artery Occlusion Stroke.” Stroke and Vascular Neurology 10: e003221. 10.1136/svn-2024-003221 PMC12107462 39164038

[brb371770-bib-0009] Huang, Y. , Z. Li , and X. Yin . 2024. “Long‐Term Survival in Stroke Patients: Insights into Triglyceride‐Glucose Body Mass Index From ICU Data.” Cardiovascular Diabetology 23: 137. 10.1186/s12933-024-02231-0 PMC11046846 38664780

[brb371770-bib-0010] Huang, Y. , X. Nie , M. Wang , et al. 2025. “Outcomes of Endovascular Thrombectomy in Acute Ischemic Stroke Patients With Insulin Resistance.” Journal of Neurointerventional Surgery 18, no. 5: 1217–1223. 10.1136/jnis-2025-023653 PMC13217024 40639940

[brb371770-bib-0011] Huo, G. , Y. Tang , Z. Liu , J. Cao , Z. Yao , and D. Zhou . 2025. “Association Between C‐Reactive Protein‐Triglyceride Glucose Index and Stroke Risk in Different Glycemic Status: Insights From the China Health and Retirement Longitudinal Study (CHARLS).” Cardiovascular Diabetology 24: 142. 10.1186/s12933-025-02686-9 PMC11948880 40140859

[brb371770-bib-0012] Jadhav, A. P. , S. M. Desai , and T. G. Jovin . 2021. “Indications for Mechanical Thrombectomy for Acute Ischemic Stroke.” Neurology 97: S126–S136. 10.1212/WNL.0000000000012801 34785611

[brb371770-bib-0013] Jayaraj, R. L. , S. Azimullah , R. Beiram , F. Y. Jalal , and G. A. Rosenberg . 2019. “Neuroinflammation: Friend and Foe for Ischemic Stroke.” Journal of Neuroinflammation 16: 142. 10.1186/s12974-019-1516-2 PMC6617684 31291966

[brb371770-bib-0014] Ji, Y. , X. Xu , K. Wu , et al. 2022. “Prognosis of Ischemic Stroke Patients Undergoing Endovascular Thrombectomy Is Influenced by Systemic Inflammatory Index Through Malignant Brain Edema.” Clinical Interventions in Aging 17: 1001–1012. 10.2147/CIA.S365553 PMC9259057 35814350

[brb371770-bib-0015] Lee, M. , C.‐H. Kim , Y. Kim , et al. 2021. “High Triglyceride Glucose Index is Associated With Poor Outcomes in Ischemic Stroke Patients After Reperfusion Therapy.” Cerebrovascular Diseases 50: 691–699. 10.1159/000516950 34229319

[brb371770-bib-0016] Li, J. , S. Jiang , P. Guo , et al. 2025. “TyG‐BMI and TyG/BMI %: Valuable Evaluation Tools for Predicting Unfavorable Prognosis in Ischemic Stroke Patients With Large Vessel Occlusion After Endovascular Therapy.” Journal of Stroke and Cerebrovascular Diseases 34: 108352. 10.1016/j.jstrokecerebrovasdis.2025.108352 40398546

[brb371770-bib-0017] Lteif, A. A. , K. Han , and K. J. Mather . 2005. “Obesity, Insulin Resistance, and the Metabolic Syndrome.” Circulation 112: 32–38. 10.1161/CIRCULATIONAHA.104.520130 15983246

[brb371770-bib-0018] Lu, Z. , Y. Xiong , X. Feng , et al. 2023. “Insulin Resistance Estimated by Estimated Glucose Disposal Rate Predicts Outcomes in Acute Ischemic Stroke Patients.” Cardiovascular Diabetology 22: 225. 10.1186/s12933-023-01925-1 PMC10464388 37633905

[brb371770-bib-0019] Ruan, G.‐T. , H.‐L. Xie , H.‐Y. Zhang , et al. 2022. “A Novel Inflammation and Insulin Resistance Related Indicator to Predict the Survival of Patients with Cancer.” Frontiers in Endocrinology 13: 905266. 10.3389/fendo.2022.905266 PMC9252441 35795140

[brb371770-bib-0020] Saltiel, A. R. , and J. M. Olefsky . 2017. “Inflammatory Mechanisms Linking Obesity and Metabolic Disease.” Journal of Clinical Investigation 127: 1–4. 10.1172/JCI92035 PMC5199709 28045402

[brb371770-bib-0021] Sun, W. , H. Shen , X. Wu , et al. 2024. “Influence of TyG Index on Large Vascular Occlusive Stroke Following Endovascular Treatment.” CNS Neuroscience and Therapeutics 30: e70143. 10.1111/cns.70143 PMC11625684 39648362

[brb371770-bib-0022] Tu, W.‐J. , B.‐H. Chao , L. Ma , et al. 2021. “Case‐Fatality, Disability and Recurrence Rates After First‐Ever Stroke: A Study From Bigdata Observatory Platform for Stroke of China.” Brain Research Bulletin 175: 130–135. 10.1016/j.brainresbull.2021.07.020 34329730

[brb371770-bib-0023] Wang, Q. , N. Liu , L. Yang , et al. 2025. “Association of Insulin Resistance Indices With Outcomes After Endovascular Treatment in Acute Basilar Artery Occlusion.” Lipids in Health and Disease 25, no. 1: 26. 10.1186/s12944-025-02828-8 PMC12853806 41366415

[brb371770-bib-0024] Yang, Y. , X. Huang , Y. Wang , et al. 2023. “The Impact of Triglyceride‐Glucose Index on Ischemic Stroke: A Systematic Review and Meta‐Analysis.” Cardiovascular Diabetology 22: 2. 10.1186/s12933-022-01732-0 PMC9825038 36609319

[brb371770-bib-0025] Yang, Y. , and A. Liu . 2025. “Associations of Cumulative Exposure and Dynamic Trajectories of the C‐Reactive Protein‐Triglyceride‐Glucose Index With Incident Stroke in Middle‐Aged and Older Chinese Adults: A Longitudinal Analysis Based on CHARLS.” Cardiovascular Diabetology 24: 386. 10.1186/s12933-025-02945-9 PMC12502363 41053803

[brb371770-bib-0026] Yeh, S.‐J. , C.‐H. Chen , Y.‐H. Lin , et al. 2023. “Serum Amyloid A Predicts Poor Functional Outcome in Patients With Ischemic Stroke Receiving Endovascular Thrombectomy: A Case Control Study.” Journal of NeuroInterventional Surgery 15: 75–81. 10.1136/neurintsurg-2021-018234 35058315

[brb371770-bib-0027] Yoo, A. J. , C. Z. Simonsen , S. Prabhakaran , et al. 2013. “Refining Angiographic Biomarkers of Revascularization.” Stroke 44: 2509–2512. 10.1161/STROKEAHA.113.001990 PMC4142767 23920017

[brb371770-bib-0028] Zhou, S. , H. Ni , H. Sang , et al. 2026. “Association Between C‐Reactive Protein‐Triglyceride‐Glucose Index and Outcomes After Endovascular Thrombectomy in Patients With Acute Ischemic Stroke.” World Neurosurgery 208: 124871. 10.1016/j.wneu.2026.124871 41698500

[brb371770-bib-0029] Zhou, Z. , J. Gao , M. Sun , H. Chen , H. Jin , and H. Ju . 2025. “Triglyceride‐Glucose (TyG) Index Combined With C‐Reactive Protein Outperforms the TyG Index Alone in Predicting Stroke in Arthritis Patients: A Nationwide Prospective Cohort Study.” Lipids in Health and Disease 24: 341. 10.1186/s12944-025-02762-9 PMC12542220 41126300

