# Supplementary material for: The C‐Reactive Protein–Triglyceride–Glucose Index and Functional Outcomes in Acute Ischemic Stroke Patients Following Endovascular Thrombectomy
Source: Brain Behav. 2026 Sep 29;16(10):e71770. doi: 10.1002/brb3.71770 (PMC13624537; doi:10.1002/brb3.71770)
Supplement: Supplementary file 1 [file BRB3-16-e71770-s001.docx]

**SUPPLEMENTARY TABLE S1.**Compared the baseline characteristics between the enrolled patients and the excluded patients.

| Characteristics | Included participants (N = 813) | Excluded participants (N = 349) | *P* value |
| --- | --- | --- | --- |
| Age, median (IQR), year | 71 (62, 76) | 71 (63, 77) | 0.278 |
| Male, n (%) | 496 (61.01) | 212 (60.75) | 0.841 |
| Admission NIHSS, median (IQR) | 13 (11, 17) | 14 (10,18) | 0.306 |
| mTICI (2b/3), n (%) | 764 (93.97) | 326 (93.41) | 0.235 |

Note: Continuous data are summarized using median (IQR), whereas categorical data are reported as n (%). Continuous variables were compared using Mann-Whitney U test, while categorical variables were analyzed with the chi-square test.

Abbreviations: NIHSS, National Institutes of Health Stroke Scale; mTICI, modified thrombolysis in cerebral infarction.

**SUPPLEMENTARY TABLE S2.** Multivariate analysis of various risk factors effected unfavorable outcome.

|  | | B | SE | aOR (95% CI) | *P* value |
| --- | --- | --- | --- | --- | --- |
|  | Age | 0.027 | 0.009 | 1.03 (1.01-1.05) | 0.003 |
|  | Sex | -0.108 | 0.208 | 0.90 (0.60-1.35) | 0.602 |
|  | History of smoking | -0.474 | 0.226 | 0.62 (0.40-0.97) | 0.036 |
|  | History of drinking | 0.022 | 0.224 | 1.02 (0.66-1.59) | 0.920 |
|  | Hypertension | 0.102 | 0.182 | 1.11 (0.78-1.58) | 0.574 |
|  | Atrial fibrillation | 0.298 | 0.198 | 1.35 (0.91-1.99) | 0.133 |
|  | Admission SBP | 0.007 | 0.004 | 1.01 (1.00-1.01) | 0.078 |
|  | Admission NIHSS | 0.079 | 0.020 | 1.08 (1.04-1.13) | < 0.001 |
|  | Occlusion site | -0.190 | 0.167 | 0.83 (0.59-1.16) | 0.267 |
|  | TOAST type | 0.150 | 0.145 | 1.16 (0.87-1.54) | 0.303 |
|  | ASPECTS | -0.225 | 0.045 | 0.80 (0.73-0.87) | < 0.001 |
|  | Collateral circulation | -0.559 | 0.131 | 0.57 (0.44-0.74) | < 0.001 |
|  | mTICI | -1.483 | 0.413 | 0.23 (0.10-0.51) | < 0.001 |
|  | TyG | 0.529 | 0.333 | 1.70 (0.88-3.26) | 0.112 |
|  | CTI | 0.372 | 0.163 | 1.45 (1.05-2.00) | 0.023 |
|  | Constant | -4.595 | 1.685 | 0.010 |  |

Note: Adjusted for age, male sex, smoking history, history of drinking , hypertension, atrial fibrillation, baseline SBP, baseline NIHSS score, occlusion site, TOAST classification, baseline ASPECTS, collateral circulation, mTICI (2b/3), and the TyG.

Abbreviations: SBP, systolic blood pressure; NIHSS, National Institutes of Health Stroke Scale; TOAST, the Trial of Org 10172 in Acute Stroke Treatment; ASPECT, Alberta Stroke Program Early CT; mTICI, modified thrombolysis in cerebral infarction; TyG, triglyceride-glucose index; CTI, C-reactive protein-triglyceride-glucose index; B, regression coefficient; SE, standard error; aOR, adjusted odds ratio.

**SUPPLEMENTARY TABLE S3** Univariate and multivariate logistic regression analyses for CTI and 90-day mortality.

| Variable | Univariate | | Multivariable | |
| --- | --- | --- | --- | --- |
|  | OR (95% CI) | *P value* | aOR (95% CI) | *P value* |
| Age | 1.06 (1.03-1.08) | < 0.001 | 1.05 (1.02-1.08) | < 0.001 |
| Male | 1.54 (1.07-2.24) | 0.022 | 1.08 (0.64-1.83) | 0.762 |
| History of smoking | 0.51 (0.33-0.79) | 0.002 | 0.56 (0.31- 1.01) | 0.054 |
| History of drinking | 0.69 (0.44-1.07) | 0.094 | - | - |
| Hypertension | 1.45 (0.97-1.16) | 0.069 | - | - |
| Diabetes mellitus | 1.32 (0.83-2.11) | 0.247 | - | - |
| Atrial fibrillation | 1.86 (1.27-2.72) | < 0.001 | 1.10 (0.66-1.85) | 0.706 |
| Baseline SBP | 1.01 (1.00-1.02) | 0.002 | 1.00 (0.99-1.01) | 0.406 |
| Baseline DBP | 1.00 (0.99-1.01) | 0.861 | - | - |
| Baseline NIHSS | 1.20 (1.14-1.25) | < 0.001 | 1.12 (1.06-1.12) | < 0.001 |
| Intravenous thrombolysis | 0.81 (0.44- 1.51) | 0.247 | - | - |
| Occlusion site | 0.40 (0.27-0.58) | < 0.001 | 0.67 (0.42-1.06) | 0.088 |
| TOAST type | 1.16 (0.87- 1.54) | 0.328 | - | - |
| Baseline ASPECTS | 0.76 (0.71-0.82) | < 0.001 | 0.81 (0.73-0.90) | < 0.001 |
| OTP (min) | 0.99 (0.99-1.00) | 0.005 | 0.99 (0.98-0.99) | < 0.001 |
| OTR (min) | 1.00 (0.99-1.00) | 0.036 | 1.01 (1.00-1.02) | < 0.001 |
| Collateral circulation | 0.424 (0.33-0.54) | < 0.001 | 0.81 (0.59-1.10) | 0.166 |
| mTICI (2b/3) | 0.22 (0.12-0.39) | < 0.001 | 0.58 (0.25-1.34) | 0.204 |
| TyG | 3.36 (2.03-5.58) | < 0.001 | 1.66 (0.74-3.71) | 0.218 |
| CTI | 2.36 (1.76-3.16) | < 0.001 | 2.18 (1.41-3.39) | < 0.001 |

Note: Adjusted for age, male sex, smoking history, hypertension, atrial fibrillation, baseline SBP, baseline NIHSS score, occlusion site, baseline ASPECTS, OTP, OTR, collateral circulation , mTICI (2b/3), and the TyG.

Abbreviations: SBP, systolic blood pressure; DBP, diastolic blood pressure; NIHSS, National Institutes of Health Stroke Scale; TOAST, the Trial of Org 10172 in Acute Stroke Treatment; ASPECT, Alberta Stroke Program Early CT; OTP, onset-to-puncture time; OTR, onset-to-reperfusion time; mTICI, modified thrombolysis in cerebral infarction; TyG, triglyceride-glucose index; CTI, C-reactive protein-triglyceride-glucose index; aOR, adjusted Odds Ratio.

**SUPPLEMENTARY TABLE S4.** Characteristics of patients with different severity of CTI.

|  | CTI quartiles | | | | |
| --- | --- | --- | --- | --- | --- |
| Characters | Q1 (N = 204) | Q2 (N = 205) | Q3 (N = 205) | Q4 (N =199) | *P* value |
| Age, median (IQR), years | 72 (64, 77) | 71 (61.5,77) | 69 (58, 75) | 70 (64, 76) | 0.212 |
| Male, n (%) | 109 (53.43) | 125 (60.98) | 142 (69.27) | 120 (60.30) | 0.013 |
| Medical history, n (%) |  |  |  |  |  |
| History of smoking, n (%) | 60 (29.41) | 64 (31.22) | 77 (37.56) | 64 (32.16) | 0.329 |
| History of drinking, n (%) | 51 (25.00) | 53 (25.86) | 61 (29.76) | 56 (28.14) | 0.693 |
| Hypertension, n (%) | 98 (48.04) | 135 (65.86) | 134 (65.37) | 151 (75.88) | <0.001 |
| Diabetes mellitus, n (%) | 16 (7.84) | 30 (14.63) | 34 (16.59) | 54 (27.14) | <0.001 |
| Atrial fibrillation, n (%) | 127 (62.25) | 103 (50.24) | 82 (40.00) | 93 (46.73) | <0.001 |
| Clinical data |  |  |  |  |  |
| Admission SBP, mean ± SD, mmHg | 149 (135, 164) | 152(140,170) | 152 (139.50, 168) | 160 (144,177) | <0.001 |
| Admission DBP, mean ± SD, mmHg | 82 (76.25, 92) | 85 (77.5, 96) | 88 (76.5, 93.5) | 86 (78, 96) | 0.150 |
| Admission NIHSS, median (IQR) | 13 (11,17) | 13 (11,16) | 13 (11,16) | 14 (12,18) | 0.004 |
| Intravenous thrombolysis, n (%) | 24 (11.76) | 25 (12.20) | 23 (11.22) | 19 (9.55) | 0.846 |
| Occlusion site, n (%) |  |  |  |  | 0.105 |
| ICA | 83 (40.69) | 78 (38.05) | 98 (47.80) | 95 (47.74) |  |
| MCA-M1 | 121 (59.31) | 127 (61.95) | 107 (52.20) | 104 (52.26) |  |
| TOAST type, n (%) |  |  |  |  | < 0.001 |
| LAA | 37 (18.14) | 69 (33.66) | 74 (36.10) | 78 (39.20) |  |
| CE | 144 (70.59) | 114 (55.61) | 102 (49.76) | 96 (48.24) |  |
| Others or Undetermined | 23 (11.27) | 22 (10.73) | 29 (14.15) | 25 (12.56) |  |
| Radiological findings |  |  |  |  |  |
| Baseline ASPECTS, median (IQR) | 8 (7, 9) | 9 (7, 10) | 8 (7, 10) | 8 (7, 9) | 0.220 |
| OTP, median (IQR), minutes | 307.5 (240, 404.5) | 316 (240, 493.5) | 320 (240, 442) | 317 (240, 503) | 0.505 |
| OTR, median (IQR), minutes | 357 (290, 457.25) | 366 (281.5, 530.25) | 390 (304, 518) | 385 (300, 570) | 0.158 |
| Collateral, n (%) |  |  |  |  | 0.158 |
| Grade 0 | 26 (12.75) | 21 (10.24) | 27 (13.17) | 38 (19.10) |  |
| Grade 1 | 52 (25.50) | 51 (24.88) | 54 (26.34) | 56 (28.14) |  |
| Grade 2 | 126 (61.76) | 133 (64.88) | 124 (60.49) | 105 (52.76) |  |
| mTICI (2b/3), n (%) | 193 (94.61) | 200 (97.56) | 188 (91.71) | 183 (91.96) | 0.044 |
| Laboratory measurements |  |  |  |  |  |
| TyG, median (IQR) | 4.37 (4.22, 4.53) | 4.54 (4.38, 4.78) | 4.67 (4.48, 4.83) | 4.85 (4.64, 5.11) | < 0.001 |
| Outcome, n (%) |  |  |  |  |  |
| unfavorable functional outcome, n (%) | 80 (39.22) | 86 (41.95) | 95 (46.34) | 124 (62.31) | < 0.001 |

Note: Continuous data are summarized using either mean ± SD or median (IQR), whereas categorical data are reported as n (%).Continuous variables were compared using independent samples t-test or Kruskal-Wallis H test, while categorical variables were analyzed with the chi-square test.

Abbreviations: CTI, C-reactive protein-triglyceride-glucose index; SBP, systolic blood pressure; DBP, diastolic blood pressure; NIHSS, National Institutes of Health Stroke Scale; ICA, internal carotid artery; MCA, middle cerebral artery; TOAST, the Trial of Org 10172 in Acute Stroke Treatment; LAA, large artery atherosclerosis; CE, cardioembolism; ASPECT, Alberta Stroke Program Early CT; OTP, onset-to-puncture time; OTR, onset-to-reperfusion time; mTICI, modified thrombolysis in cerebral infarction; TyG, triglyceride-glucose index; CTI, C-reactive protein-triglyceride-glucose index; SD, standard deviation; IQR, interquartile range.

**SUPPLEMENTARY TABLE S5.** Multivariable logistic regression for functional outcome using a stricter univariate screening threshold (*P* < 0.01).

|  | | B | S.E | aOR (95%CI) | *P* value |
| --- | --- | --- | --- | --- | --- |
|  | Age | 0.027 | 0.009 | 1.03 (1.01-1.05) | 0.003 |
|  | Sex | -0.103 | 0.202 | 0.90 (0.61-1.34) | 0.609 |
|  | History of smoking | -0.466 | 0.212 | 0.63 (0.41-0.95) | 0.028 |
|  | Hypertension | 0.103 | 0.182 | 1.11 (0.78-1.58) | 0.572 |
|  | Atrial fibrillation | 0.299 | 0.198 | 1.35 (0.91-1.99) | 0.131 |
|  | Admission SBP | 0.007 | 0.004 | 1.01 (1.00-1.01) | 0.077 |
|  | Admission NIHSS | 0.079 | 0.020 | 1.08 (1.04-1.13) | < 0.001 |
|  | Occlusion site | -0.190 | 0.171 | 0.83 (0.59-1.16) | 0.267 |
|  | TOAST type | 0.150 | 0.145 | 1.16 (0.87-1.55) | 0.302 |
|  | Baseline ASPECTS | -0.225 | 0.045 | 0.80 (0.73-0.87) | < 0.001 |
|  | Collateral circulation | -0.559 | 0.131 | 0.57 (0.44-0.74) | < 0.001 |
|  | mTICI (2b/3) | -1.482 | 0.413 | 0.23 (0.10-0.51) | < 0.001 |
|  | TyG | 0.531 | 0.333 | 1.70 (0.89-3.26) | 0.110 |
|  | CTI | 0.371 | 0.163 | 1.45 (1.05-2.00) | 0.023 |
|  | Constant | -4.599 | 1.684 | 0.10 | 0.006 |

Note: adjusted for age, sex, smoking history, hypertension, atrial fibrillation, baseline SBP, baseline NIHSS score, occlusion site, TOAST classification, baseline ASPECTS, collateral circulation, mTICI (2b/3), and the TyG.

Abbreviations: SBP, systolic blood pressure; NIHSS, National Institutes of Health Stroke Scale; TOAST, the Trial of Org 10172 in Acute Stroke Treatment; ASPECT, Alberta Stroke Program Early CT; mTICI, modified thrombolysis in cerebral infarction; TyG, triglyceride-glucose index; CTI, C-reactive protein-triglyceride-glucose index; B, regression coefficient; SE, standard error; aOR, adjusted Odds Ratio.

**SUPPLEMENTARY FIGURE S1**. ROC curve for CTI to predicting 90-day unfavorable functional outcome.

Note: The area under the ROC curve (AUC) of CTI was 0.594 (95% CI 0.554-0.663, *P* < 0.001)

Abbreviations: AUC, area under the ROC curve.

**SUPPLEMENTARY FIGURE S2.** ROC curve comparison of Model A versus Model B by DeLong test to predicting 90-day unfavorable functional outcome.

Note: Model A included gender, age, smoking history, alcohol consumption, hypertension, atrial fibrillation, admission SBP, baseline NIHSS, occlusion site, TOAST classification, ASPECTS, collateral circulation status, mTICI (2b/3), TyG and CTI. Model B included gender, age, smoking history, alcohol consumption, hypertension, atrial fibrillation, admission SBP, baseline NIHSS, occlusion site, TOAST classification, ASPECTS, collateral circulation status, mTICI (2b/3) and CTI, without TyG index.

Abbreviations: AUC, area under the ROC curve.
